# Facing Danger: Exploring Personality and Reactions of European Hedgehogs (*Erinaceus europaeus*) towards Robotic Lawn Mowers

**DOI:** 10.3390/ani14010002

**Published:** 2023-12-19

**Authors:** Sophie Lund Rasmussen, Bettina Thuland Schrøder, Anne Berger, David W. Macdonald, Cino Pertoldi, Elodie Floriane Briefer, Aage Kristian Olsen Alstrup

**Affiliations:** 1 Wildlife Conservation Research Unit, The Recanati-Kaplan Centre, Department of Biology, University of Oxford, Tubney House, Abingdon Road, Tubney, Abingdon OX13 5QL, UK; david.macdonald@biology.ox.ac.uk; 2 Department of Chemistry and Bioscience, Aalborg University, Fredrik Bajers Vej 7H, 9220 Aalborg, Denmark; cp@bio.aau.dk; 3 Linacre College, University of Oxford, St. Cross Road, Oxford OX1 3JA, UK; 4 Behavioral Ecology Group, Section for Ecology and Evolution, Department of Biology, University of Copenhagen, Universitetsparken 15, 2100 Copenhagen, Denmark; catsandconservation@gmail.com (B.T.S.); elodie.briefer@bio.ku.dk (E.F.B.); 5 Leibniz Institute for Zoo and Wildlife Research, Alfred-Kowalke-Straße 17, 10315 Berlin, Germany; berger@izw-berlin.de; 6 Aalborg Zoo, Mølleparkvej 63, 9000 Aalborg, Denmark; 7 Department of Nuclear Medicine and PET, Aarhus University Hospital, Palle Juul-Jensens Boulevard 165, 8200 Aarhus, Denmark; aagealst@rm.dk; 8 Department of Clinical Medicine, Aarhus University, Palle Juul-Jensens Boulevard 165, 8200 Aarhus, Denmark

**Keywords:** *Erinaceus europaeus*, animal personality, applied animal behaviour research, shyness–boldness, wildlife conservation, anthropogenic disturbance, robotic lawn mowers, garden technology, lawn care, behavioural instability

## Abstract

**Simple Summary:**

The European hedgehog is a generally welcomed but nowadays less common guest in residential gardens, as the species is in decline. Sharing habitats with humans comes at a cost: a residential garden holds many potential dangers for hedgehogs. Previous research has shown that certain models of robotic lawn mowers may harm hedgehogs. This study sought to investigate the personality and reactions of live hedgehogs towards a disarmed, approaching robotic lawn mower. Personality tests revealed that the hedgehogs could be divided into categories of “shy” and “bold” individuals, independently of age and sex. The encounter tests with a disarmed robotic lawn mower showed that they behaved and positioned themselves in seven different ways, and the individuals with a bold personality reacted in a more unpredictable way. Adult hedgehogs tended to react in a shyer manner, and the tested hedgehogs, generally, acted less boldly the second time they encountered a robotic lawn mower. This knowledge will be used in the process of designing a standardised hedgehog safety test to eventually produce and approve hedgehog-friendly robotic lawn mowers that pose no hazards to hedgehogs, ultimately, serving to eliminate their influence on hedgehog survival and, thereby, improve hedgehog conservation.

**Abstract:**

The populations of European hedgehog (*Erinaceus europaeus*) are in decline, and it is essential that research identifies and mitigates the factors causing this. Hedgehogs are increasingly sharing habitats with humans, being exposed to a range of dangers in our backyards. Previous research has documented that some models of robotic lawn mowers can cause harm to hedgehogs. This study explored the personality and behaviour of 50 live hedgehogs when facing an approaching, disarmed robotic lawn mower. By combining a novel arena and novel object test, we found that 27 hedgehogs could be categorised as “shy” and 23 as “bold”, independently of sex and age. The encounter tests with a robotic lawn mower showed that the hedgehogs positioned themselves in seven different ways. Personality did not affect their reactions. Adult hedgehogs tended to react in a shyer manner, and the hedgehogs, generally, acted less boldly during their second encounter with the robotic lawn mower. Additionally, our results show that bold individuals reacted in a more unpredictable way, being more behaviourally unstable compared to the shy individuals. This knowledge will be applied in the design of a standardised hedgehog safety test, eventually serving to produce and approve hedgehog-friendly robotic lawn mowers.

## 1. Introduction

The European hedgehog (*Erinaceus europaeus*), hereafter referred to as “hedgehog”, is a widely distributed species that can survive across a range of diverse habitat types [1,2]. Despite the species’ ability to adapt to many different settings [3], recent research has either documented or suggested declines in the populations of European hedgehogs in several Western European countries [4,5,6,7,8,9,10,11,12,13,14]. For the time being, the investigated factors contributing to this decline include habitat loss; habitat fragmentation; inbreeding; intensified agricultural practices; road traffic accidents; a reduction in biodiversity and, hence, food items; lack of suitable nest sites in residential gardens; accidents caused by garden tools; netting and other anthropogenic sources in residential gardens; infections with pathogens and endoparasites; molluscicide and rodenticide poisoning; and, in some areas, badger predation [3,5,15,16,17,18,19,20,21,22,23,24,25,26,27,28,29,30,31]. With the potential to reach 16 years of age [32], it is of concern that the majority of research into the lifespan of European hedgehogs has found a mean age of only two years (see Rasmussen, Berg, Martens and Jones [32], Table 1 for an overview). It is, therefore, essential to investigate the possible causes for this early mortality and the overall population decline in order to optimise the conservation initiatives directed at this species.

### 1.1. Hedgehogs and Robotic Lawn Mowers

Robotic lawn mowers are becoming increasingly popular in Europe and the US. According to market insight reports, it is expected that the global robotic lawn mower market will expand from US 0.8–1.5 billion in 2020–2022 to US 2.7–4 billion in 2032, and the market is anticipated to develop at a compound annual growth rate (CAGR) of 11.5–15.5% during the forecasted period [33,34]. With the increasing number of robotic lawn mowers operating in residential gardens throughout the distribution range of hedgehogs in Europe, combined with the fact that research indicates that hedgehogs are, nowadays, increasingly associated with human-occupied areas [10,20,31,35], it seems likely that many individual hedgehogs will encounter several robotic lawn mowers during their lifetime. 

Human activities negatively impact the welfare of countless wild vertebrates [36], and hedgehogs are no exception. Injured hedgehogs are frequently found by members of the public and are admitted to hedgehog rehabilitation centres with different types of cuts and injuries, most often caused by garden tools and netting, or predators such as dogs, foxes, or badgers [37,38,39,40]. In some cases, the injuries are fatal, whilst others necessitate euthanasia. However, because of a growing concern reported by hedgehog carers and members of the public that an increasing number of these incidents could have been caused by robotic lawn mowers, Rasmussen et al. (2021) [29] investigated the effects of robotic lawn mowers on hedgehogs. Tests were carried out on deceased hedgehogs and showed that some models of robotic lawn mowers did, indeed, injure hedgehog carcasses, whereas other models only had to touch a hedgehog carcass lightly to detect it before changing direction accordingly to avoid it. Consequently, it was observed that all models of robotic lawn mowers included in the tests had to physically interact with the hedgehog carcasses to detect them [29]. This led to the suggestion that research should be initiated to develop more hedgehog-friendly robotic lawn mowers and that a standardised safety test should be designed for the evaluation and approval of new models of robotic lawn mowers for the market in terms of hedgehog safety as an addition to the current mandatory general safety guidelines [29,41]. This test should, ideally, be performed on a specially designed hedgehog crash test dummy placed in realistic fixed positions.

Having previously tested the effects of robotic lawn mowers on dead hedgehogs, positioning the carcasses in the tests based on the knowledge of hedgehog behaviour, we decided to test the reactions of live hedgehogs to an approaching robotic lawn mower to optimise the design of a future standardised safety test by ensuring that it is realistic. Furthermore, testing and quantifying the behaviour of live animals should also consider the effects of personality on the outcome. 

### 1.2. Measuring Personality in Animals

When facing danger, hedgehogs tend to stand completely still, as if they are in a frozen state, often in an upright position with the snout pointing inwards (i.e., head bent inwards in a partially curled-up position, later referred to as behavioural category 3), whilst deciding whether the next step should be to curl up or run away [42]. The strategy of curling up in front of an approaching car or robotic lawn mower appears to be less successful than running away. Could personality determine the reaction of hedgehogs towards an approaching robotic lawn mower? 

Personality, defined as individual differences that are stable over time and across situations, affects how individuals react to challenging situations [43] and may influence their survival [44]. Several studies have shown that it is possible to estimate the shyness/boldness of individuals, including hedgehogs [45], by analysing how they explore a novel environment or arena, or by measuring their latency to approach a novel object in a familiar environment [44,46,47,48]. Previous research has explored and documented the occurrence of a shyness–boldness gradient in natural populations [44,49,50,51]. In addition, it has been demonstrated that personality potentially influences fitness through reproductive success and survival [44,52,53]. Natural selection affects factors such as boldness at a population level [54], which is why individuals with inappropriate levels of boldness may suffer reduced fitness in the wild due to extensive risk-taking behaviour [44]. Accordingly, Bremner-Harrison, Prodohl, and Elwood (2004) [44] found that bolder juvenile captive-bred swift foxes had a lower postrelease survival compared to their shyer conspecifics. 

Another important aspect that characterises the personality of an individual is the degree of behavioural instability, which quantifies the degree of unpredictability of a behavioural response. The notion of behavioural instability, as suggested by Pertoldi et al. (2016) and others [55,56,57], goes beyond being solely described by the variance and/or interquartile range (IQR). It also encompasses the kurtosis and skewness (i.e., asymmetry) of the distributions. These parameters collectively impact the median absolute deviation, a measure of the variability in a dataset, which is estimated by the median distance of the data values from the median (MAD). The higher levels of behavioural instability exhibited by certain individuals could have relevant implications, as an increased variability in the behavioural repertoire could enhance the probability of survival in a dangerous situation, such as an encounter between a hedgehog and a robotic lawn mower [55].

### 1.3. Aim of the Research

In this study, we tested the reactions of live hedgehogs towards a disarmed, approaching robotic lawn mower to optimise the test design of a future standardised realistic safety test. Additionally, we investigated the effects of personality, measured as shyness/boldness, on behavioural responses and associated predictability and, thereby, the risk-taking behaviour of these animals when facing the approaching robotic lawn mower to ensure that this test would account for differences in the hedgehog reactions linked to their personality. We predicted that shy individuals would have a higher tendency to run away and that bolder individuals would be more inquisitive towards the approaching robotic lawn mower.

Measuring the responses of live hedgehogs towards an approaching robotic lawn mower will facilitate the optimal design of a standardised safety test for robotic lawn mowers, which will become an important tool for enhancing the safety of hedgehogs entering gardens. This will, ultimately, result in the improved conservation of the declining populations of European hedgehogs. 

## 2. Materials and Methods

**Figure 1:**
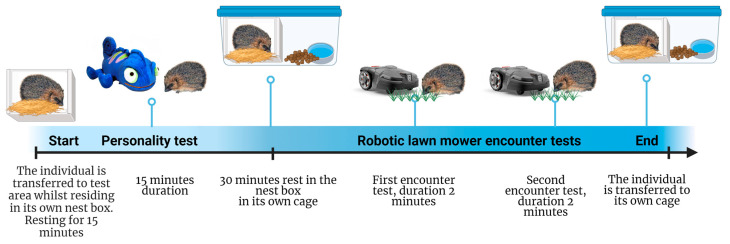
Experimental design and timeline. Design adapted from the “Mouse Experimental Timeline”, template by BioRender.com, agreement number: RS262OWRC1. Photographs and illustrations by Cloud b; Encyclopaedia Britannica, and Husqvarna.

The research included both personality tests and encounter tests with a robotic lawn mower (Figure 1), performed on 50 live rehabilitated Danish hedgehogs that had been assessed as ready for release back into the wild. All individuals were released at suitable sites within a few days after the tests. Some individuals were adults originally admitted into care because of disease or injury (N = 15), and some were orphaned juveniles (N = 35) that were hand-raised by a hedgehog rehabilitator. They had reached the age and capabilities similar to wild juveniles at the age of independence, having been raised under natural conditions, and had been deemed ready for release back into the wild. 

The experimental design was created with the welfare of the hedgehogs in mind, taking precautions to reduce the levels of stress caused by transportation and handling. Therefore, all tests took place in the garden surrounding the wildlife rehabilitation centre, where the hedgehogs had been in care, and the hedgehogs were exclusively handled by the wildlife rehabilitator, who had been nursing them back to health.

The tests were divided into a pilot study on ten individuals, which took place on two consecutive nights from the 3rd–5th of June 2022, and further tests on 40 individuals taking place from the 6th–8th of September 2022 (N = 20) and 15th–17th of September 2022 (N = 20). As hedgehogs are nocturnal, the experiments were performed during their natural activity period, from sunset to sunrise, varying based on the time of year and the number of individuals tested on the specific nights of testing. 

### 2.1. Personality Tests

The personality tests were performed as a combination of a novel object test [48,58,59] and a novel arena test [59,60] for the purpose of assessing each individual’s tendency to respond with either curiosity or fearfulness and, hence, exploration or avoidance of the novel object in a novel environment. The novel object was a 45 × 25 × 20 cm blue plush toy (Cloud b “Charley the Chameleon”). The toy stimulated both visual and auditory senses through a sound emission, a soothing melody at a maximum of 5 dB, and blinking RGB LED lights in the colours red, blue, and green. 

The novel arena consisted of a wooden pallet frame with a solid, wooden pallet base (dimensions inside the arena: length 104 cm, width 76 cm, and height 38 cm). The base was covered by a large piece of cardstock thick paper in a light grey colour, covering the whole base of the arena. The novel object was placed in the end of the test arena, 60 cm from the nest box containing the hedgehog subjected to the personality test, which was placed in the opposite end of the novel arena. The nest box was added to the novel arena just before the test started. The novel arena was placed under a cover by a dark-blue garden gazebo for the purpose of standardising the weather conditions and shielding the arena against rain. 

**Figure 2:**
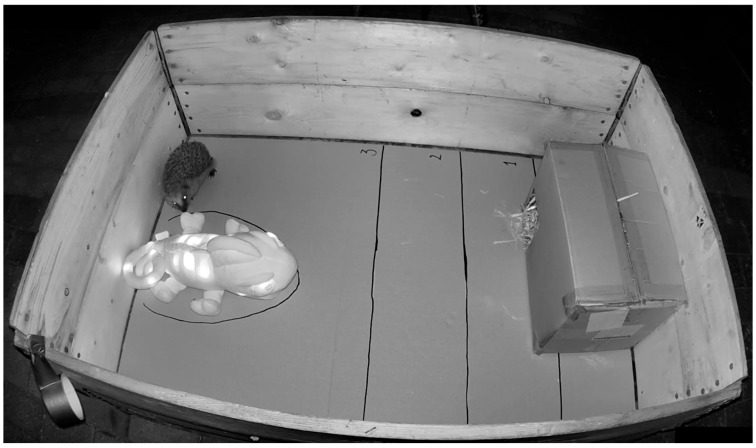
A photo from the Ring surveillance camera, which was used during the personality tests, illustrating the experimental design.

The procedure for each personality test lasted for 30 min (including rest) in total (Figure 1). Before the test started, the hedgehog was transported to the novel arena in its individual, familiar nest box, from its own enclosure situated in the housing ward of the hedgehog rehabilitation centre. The closed nest box was placed in the novel arena, allowing the hedgehog to rest and acclimatise for 15 min after transportation. During this resting period, the novel object was not activated and was not visible to the hedgehog. After 15 min of rest, the nest box entrance was opened, and the novel object’s light and sound effects were switched on (Figure 2). Over the following 15 min, two cameras recorded any activity in the novel arena. A Ring Stick Up Cam video camera (Ring^TM^, Santa Monica, CA, USA), which was suspended from the ceiling of the garden gazebo at around the centre of the novel arena, provided a complete overview of the novel arena. The Ring camera was controlled through the Ring app and was set to record with a live view continuously for 12 min at a time, as this was the maximum possible duration per recording. The Ring camera was manually re-activated after 12 min using the Ring app. A Nedis HD Wildlife CameraWCAM130GN (Nedis, ’s-Hertogenbosch, The Netherlands) was also set to record for 30 s after the detection of any locomotion and to record back to back with no delay between recordings. The Nedis camera was placed on a tripod halfway along the long side of the novel arena allowing for all activity during a test to be detected and recorded. 

An experimenter (the same person for all personality tests) silently monitored all tests in complete darkness at a distance of 4 m from the test arena. 

### 2.2. Encounter Tests with a Robotic Lawn Mower

**Figure 3:**
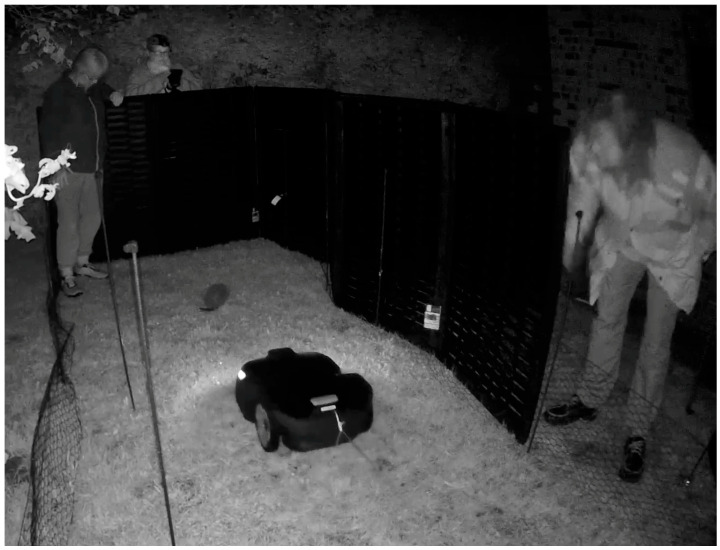
A photo from the surveillance camera used during the encounter tests, illustrating the experimental design. A range of safety precautions for the tests can be seen: (1) a person standing on the right side of the test area stepping in to manually turn off the robotic lawn mower by pushing the (visual) button on the shield of the mower; (2) the rope attached to the back of the mower (with a person pulling it in the other end); and (3) the hedgehog rehabilitator standing inside the test area next to the hedgehog ready to intervene, if necessary. The two persons standing in the vicinity of the hedgehog tested (the hedgehog rehabilitator and the person recording with the handheld FLIR E54 Thermal Imaging camera) remained the same throughout all tests and were cautious to behave in the same manner during all tests.

The robotic lawn mower encounter tests were conducted on the lawn of the residential garden surrounding the wildlife rehabilitation centre, where the hedgehogs were housed. The experimental area was fenced off with a green, 45 cm tall wire mesh fence to ensure the hedgehogs tested could not run away (Figure 3). The area was 1.5 m in width and 6.75 m in length. Green tentor poles were placed alongside the fence inside the test area and marked each 1 m distance from the hedgehog. A black folding fence, which was 1.2 m in height, consisting of three panels that were 0.8 m in width, was placed outside the fenced area to surround the zone in which the hedgehog was placed. This allowed for the experimenters recording the behaviour and stepping into the test zone to manually stop the robotic lawn mower and to remain visually hidden from the hedgehog during the tests. 

The robotic lawn mower (Husqvarna Automower^®^ 415X, Husqvarna, Huskvarna, Sweden) was positioned on a spot marked with duct tape at a distance of 5.75 m from the hedgehog, allowing it to move in a straight line through the test area in the direction of the hedgehog. At the beginning of each test, the hedgehog was placed inside the fenced area, on a spot on the lawn marked with duct tape, at a distance of 1 m from the surrounding fence. The hedgehog was positioned in an upright standing position with the front facing the mower and the head folding inwards (later referred to as behavioural category 3). When the hedgehog was in place, the mower was turned on, causing it to move in the direction of the hedgehog and gradually approaching the individual at 380 mm/s, until a distance of 0.5 m, at which the mower was stopped. During the pilot test on the first ten individuals, the robotic lawn mower was stopped at a 1 m distance from the hedgehogs, but this was changed to 0.5 m for the tests with the last 40 individuals due to a concern that the 1 m distance was too far away to properly trigger a reaction from the hedgehogs.

Each hedgehog was tested with the robotic lawn mower twice: one test in which the headlights of the mower were switched off and another with the headlights switched on. The second test was performed immediately after the first (Figure 1). The order of the tests was counterbalanced among the individuals (Figure 1). In the case that the test was interrupted because of a technical failure, or if the hedgehog ran away before the robotic lawn mower was turned on, we restarted the test once, resulting in a maximum of three tests and a duration of a maximum of 15 min in total, during which the hedgehog was situated in the test area. In the majority of cases, we only tested the hedgehogs twice. 

The tests were recorded with a Ring Stick Up Cam video camera placed on a tripod overlooking the experimental area, as well as a handheld FLIR E54 Thermal Imaging camera (Teledyne FLIR, Wilsonville, OR, USA) recording from behind the folding fence surrounding the hedgehog being tested. With the use of these cameras, the reactions (i.e., behavioural categories) of each individual towards the approaching robotic lawn mower during the two tests, were recorded. 

After the tests, the hedgehog was returned to its enclosure in the housing ward at the hedgehog rehabilitation centre by the hedgehog rehabilitator.

#### Safety Precautions during the Encounter Tests

Multiple initiatives were implemented to ensure that the safety measures were met during the tests. The pivoting blades (i.e., knives) were removed from the robotic lawn mower, and the mower was controlled via remote control (Husqvarna Automower^®^ Connect App, Husqvarna, Huskvarna, Sweden). Additionally, a rope was fastened to the robotic lawn mower, and a staff member held this rope during the tests, generating manual control to ensure the mower never got closer to the hedgehog than the 0.5 m (1 m in the pilot study) distance allowed in the test. Furthermore, a person standing alongside the test area, hidden behind the folding fence, stepped in to stop the robotic lawn mower manually when it reached the 0.5 m (1 m in pilot study) limit by pressing the red stop button on the dorsal shield of the robotic lawn mower. The hedgehog carer remained standing next to the hedgehog during the tests and was ready to intervene and pick up the hedgehog in case it tried to run away or the other safety precautions failed (which never occurred during the tests).

### 2.3. Data Analysis

The data analyses and graphs for publication were prepared in R v 4.2.3. [61] using the packages lme4, DHARMa, pbkrtest, multcomp, ordinal, car, and tidyverse. 

#### 2.3.1. Personality Tests

On the basis of their behaviour during the combined novel arena and novel object test, the hedgehogs were categorised as either shy or bold as follows: individuals that remained in their nest box during the full 15 min duration of the test were categorised as “shy”, whilst those that left the nest box and entered the arena to explore the novel arena and novel object during the test were categorised as “bold”. This categorisation was established during the pilot study after observing a large number (6/10) of hedgehogs remaining in their nest box for the entire test.

We used a generalised linear model (GLM) to analyse the results of the personality tests due to a binary outcome (shy versus bold, analysed as 0 vs. 1) and having no repeated measures of the same individuals. The GLM with binomial family included personality (0 versus 1) as a response variable and age (adult versus juvenile), sex (female versus male), and weight (in kg, z-score transformed) as fixed effects. 

On the basis of the variance inflation factor (VIF), using the VIF function in R, testing for multicollinearity, we found that the fixed effects of age and weight were correlated, as VIF > 5. Therefore, we removed the response variable “weight” from the initial model (VIF = 5.42). Accordingly, the final model was: glm (personality_category ~ sex + age, family = binomial, data = personality). 

#### 2.3.2. Encounter Tests with a Robotic Lawn Mower

During the encounter tests with a robotic lawn mower, the behaviour of each individual was scored in five ordinal behavioural categories, with 1 being the more exploratory behavioural (bold) reaction and 5 being the more cautious (shy) reaction. Behaviour was recorded at the point in time at which the robotic lawn mower was situated just in front of the hedgehog and before the mower was manually stopped. 

The ordinal categories were:

(1) Upright position with snout facing 9–10 o’clock, 2–3 o’clock, or 6 o’clock (rump towards the mower).
(2) Upright position with snout facing the mower.
(3) Upright position with snout pointing inwards (head bent inwards in a partially curled-up position). This was the position the hedgehog was originally placed in when the test was initiated.
(4) Hedgehog running away from the robotic lawn mower.
(5) Hedgehog curling up.

To analyse the results of the encounter tests, a cumulative link mixed model (CLMM) was chosen, as the response is ordinal (ordered categorical data), and no assumption was made concerning the spacing between behavioural categories 1 and 5. 

The CLMM included the ordinal behavioural category (1–5) as a response variable; lights (on vs. off), personality (shy vs. bold, coded as 0 vs. 1), age (adult versus juvenile), sex (female vs. male), and test number (1 vs. 2 as a control) as fixed effects; and individual ID crossed with the date of the test as random effects (to control for repeated measures, as each hedgehog was tested twice, and the differences between the days). The variance inflation factor was below 5 for all fixed effects, indicating the absence of multicollinearity, and allowing us to include all factors in the same model. The final model was: clmm (responseF ~ lights + personality_cat + weight + sex + test_number + (1|individual) + (1|date), data = behaviour). 

Furthermore, by comparing a model with to a model without the random effect “individual” using a likelihood ratio test, we tested whether individuals differed in their responses in general. Finally, the degree of behavioural instability, estimated as the median absolute deviation (MAD) of the ordinal behavioural category was tested with the Mann–Whitney U test and Levene’s test from the medians for differences among the individuals belonging to the two categories of personality (shy vs. bold). In the two categories of personality, individuals of different sex, weight, and age were pooled, as these factors did not seem to affect personality (see Section 3, results below). 

### 2.4. Ethical Approval

Ethical approval for this study was provided by the Animal Experiments Inspectorate under the Danish National Committee for the Protection of Animals used for Scientific Purposes (license number: 2021-15-0201-00865) in accordance with 2010/63/EU [62]. We followed the 3R concept for use of animals in research: we used a paired design to reduce the number of experimental animals needed (i.e., reduction), and we ensured that no animal suffered harm during the study through specified safety measures (i.e., refinements). It was not possible to replace the studies with alternative methods (i.e., replacement). Permission was also obtained from the Danish Nature Agency to work with this protected species. All animals completed the tests without injury and were released back into the wild within a few days.

## 3. Results

The 50 individuals tested comprised 15 adults and 35 independent juveniles, 30 females and 20 males. 

### 3.1. Personality Tests

On the basis of their behaviour during the combined novel arena and novel object test, 27 (54%) individuals were categorised as shy and 23 (46%) as bold. 

Our data analysis showed that neither sex (GLM: Z = −0.93, *p* = 0.35) nor age (and thereby weight) (GLM: Z = 1.31, *p* = 0.19) had a significant effect on the personality of the hedgehogs (see Appendix A for the raw data). 

### 3.2. Encounter Tests with a Robotic Lawn Mower

**Figure 4:**
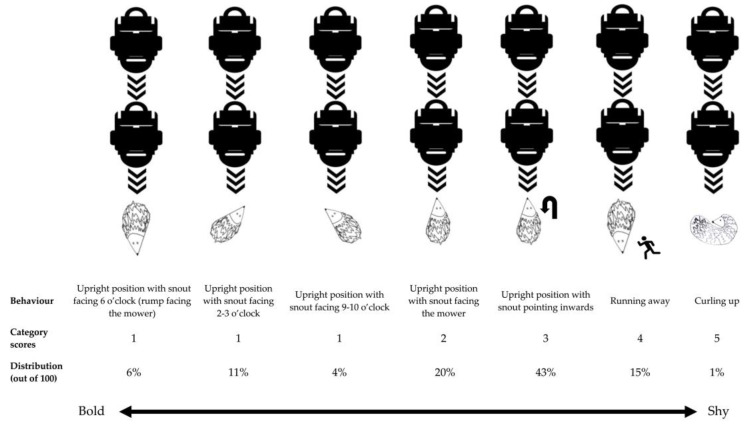
An overview of the results from the encounter tests with a robotic lawn mower. “Behaviour” describes the behaviour exhibited by the hedgehogs. “Category scores” indicate the degree of shyness or boldness of the behaviour, with 1 being the boldest and 5 being the shyest. “Distribution” denotes the frequency of the specific behaviours, in percentage, out of 100 tests conducted with a total of 50 different hedgehogs. The line with arrows indicates the gradient of shyness or boldness of the behaviour exhibited by the hedgehogs tested. At the beginning of each test, the hedgehog was placed in an “upright position with snout pointing inwards”.

The distribution of the behaviours shown by the 50 individuals tested during the total 100 encounter tests with a robotic lawn mower is illustrated in Figure 4 (see Appendix A for a full overview of the results). The most frequent behavioural response (43%) was the upright position with the snout pointing inwards, which was also the position the hedgehogs were placed in when the tests started. In 15% of the cases, the strategy of the hedgehogs was to run away. Curling up was only observed once in the 100 tests that were conducted. 

**Figure 5:**
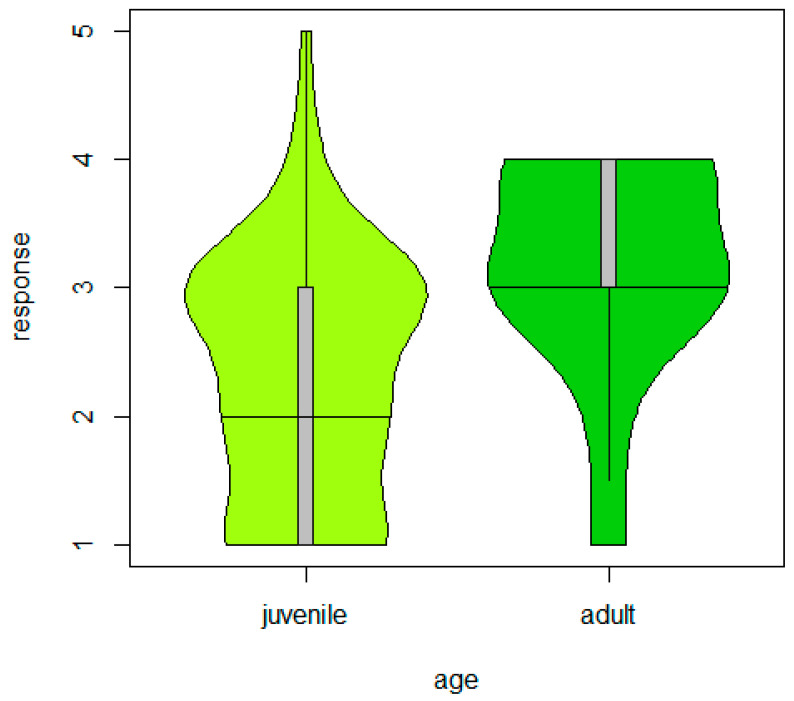
Behavioural responses as a function of the age category of the individuals during the encounter test with a robotic lawn mower (CLMM: *p* = 0.0002). “Response” shows behavioural categories 1–5, with 1 being the most inquisitive (i.e., bold) and 5 being the most timid (i.e., shy) reactions. Violin and box plots: the horizontal line shows the median, with the box extending from the lower to the upper quartiles and the whisker to the data extremes.

**Figure 6:**
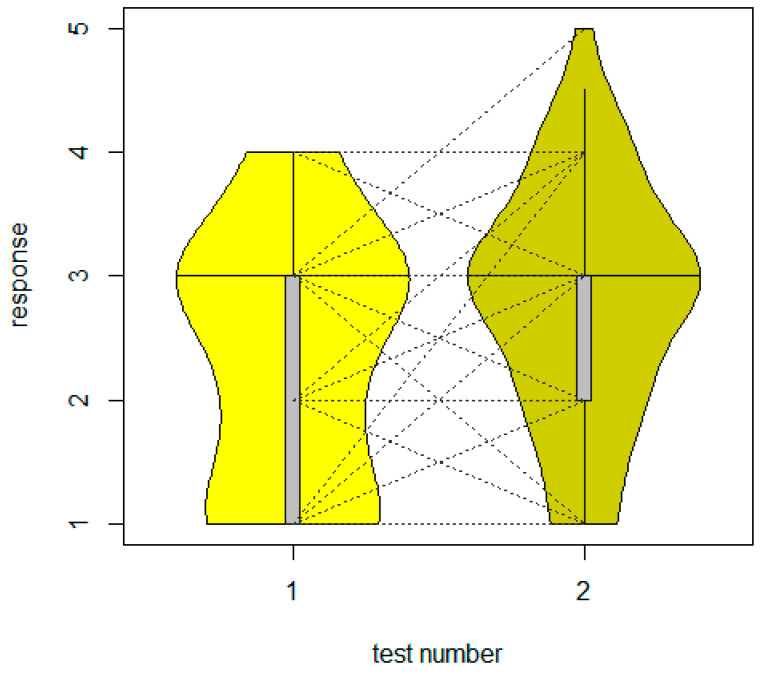
Behavioural responses of the individuals during the first and the second encounter tests with a robotic lawn mower (CLMM: *p* = 0.021). “Response” indicates the behavioural categories 1–5, with 1 being the boldest and 5 being the shyest. Violin and box plots: the horizontal line shows the median, with the box extending from the lower to the upper quartiles and the whiskers to the data extremes. The dashed lines indicate repeated measures (i.e., connecting responses by the same individual between tests 1 and 2).

We found that age had an effect on the behavioural responses of the hedgehogs (CLMM: Z = −3.71, *p* = 0.0002), with younger and, thereby, lighter individuals showing a bolder behavioural response to the approaching robotic lawn mower compared to the older and heavier individuals (Figure 5). In addition, the behavioural responses were also affected by the test number (CLMM: Z = 2.32, *p* = 0.021), with hedgehogs reacting in a shyer manner during the second of the two encounter tests with the robotic lawn mower, regardless of whether the lights were turned on or off during the first test (Figure 6). The other fixed factors (lights on/off, personality, and sex) did not reach a low *p*-value (CLMM: *p* ≥ 0.54 for all).

The random effect “individual” did not significantly affect the behavioural responses, indicating an absence of clear differences among subjects (CLMM: χ^2^ = 0.50, df = 1, *p* = 0.48; Figure 6). 

The behavioural instability of the ordinal behavioural category estimated with the MAD showed a significantly higher variability in the individuals belonging to the bold category compared to the individuals belonging to the shy category (MAD bold > MAD shy, Mann–Whitney *U*-test; H = 5.26; *p* = 0.022 Levene’s test: *p* < 0.0029).

## 4. Discussion

Our research showed that the 50 hedgehogs tested could be categorised as shy and bold, regardless of sex and age. Personality was not found to influence their reaction towards an approaching robotic lawn mower. However, we observed a higher level of behavioural instability exhibited by the bold individuals. During the encounter tests, seven different behavioural reactions were observed in the hedgehogs. There was a tendency for adults and, therefore, more experienced individuals to behave in a more eluding manner when facing an approaching robotic lawn mower. The hedgehogs, in general, acted less boldly during their second encounter test. This could be important from a conservation and welfare perspective if the experience gained from an encounter with a robotic lawn mower could reduce the risk of this individual coming into contact with such a potential risk in the future.

### 4.1. Distribution of Personality and Behavioural Instability

We found that 54% (N = 27/50) of the individuals could be categorised as shy, as they did not exit their nest box during the whole test, whilst the rest (46%; N = 23/50) could be categorised as bold. Neither sex nor weight (and, thereby, age) had an effect on personality. This is in line with the suggestion that individuals in a given population can be divided into a shy–bold continuum across age classes [48]. The findings of a balanced distribution of shy and bold individuals in the test group are also supported by the current lack of evidence for the pace-of-life syndrome (POLS) in populations of hedgehogs. The POLS predicts that behavioural traits such as high boldness, exploration, aggressiveness, or activity increase the acquisition of resources at the expense of life span, causing individuals expressing these traits to exhibit a faster life history, i.e., a higher growth rate compared to other conspecifics [45,63,64,65].

However, the higher levels of behavioural instability in the bold individuals could have some relevant implications, as a higher variability in the behavioural repertoire could enhance the probability of survival when an individual encounters a dangerous situation, as proposed by Pertoldi et al. (2016) [55], which suggests that heightened behavioural instability could possess adaptive value in an unpredictable environment. If a higher variability in the behavioural repertoire can increase the probability of surviving an encounter with a robotic lawn mower, then bold individuals could be favoured.

### 4.2. Insight Gained from the Encounter Tests with a Robotic Lawn Mower

After the 100 encounter tests performed with 50 live hedgehogs exposed to an approaching robotic lawn mower, it became evident that the reactions of the hedgehogs could be divided into seven different behavioural categories, resulting in six different test positions (excluding the behaviour of running away). These positions adopted by the hedgehogs in reaction to the robotic lawn mower will be applied in a test design with the purpose of describing the safety and effects of particular models of robotic lawn mowers on hedgehogs, using both dead hedgehogs and hedgehog crash test dummies to provide realistic test scenarios for the standardised safety tests. 

Interestingly, we found that the tested individuals tended to behave in a more timid manner during the second trial. This could indicate that the hedgehogs adjusted their behaviour towards the robotic lawn mower based on their experience, which might imply that they learned to avoid future encounters with the robotic lawn mower after a single undramatic episode. If this is, indeed, the case in real life, this could potentially prove very beneficial to the survival of hedgehogs, provided that they are not critically harmed in an encounter with a robotic lawn mower. In testing for differences in the behavioural responses among the individuals, in general, no significant effects were found. This suggests that there were no marked differences in the responses among the individuals and/or that each individual might have reacted differently in the two encounter tests. This is in line with the findings that the hedgehogs reacted in a manner that was more timid during the second of the two encounter tests with the robotic lawn mower. Similar results were found, for example, in rainbow trout, whereby boldness was reduced as an adaptive response to negative experiences in previous tests [47]. 

Older individuals exhibited shyer behaviour in the encounter tests with the robotic lawn mower. We suggest that this could be due to experience and that the independent juveniles we tested were, in general, more naïve because of their young age and lack of experience. The independent juveniles we tested (N = 35/50 individuals in total) had been hand raised in captivity. They, hence, likely had never encountered a robotic lawn mower before. Unfortunately, we have no knowledge of whether the adults (N = 15/50) included in the tests had had any previous confrontations with robotic lawn mowers in the wild before they were admitted into care. This finding could suggest that there should be a focus on the size/age category of independent juveniles in the experimental design of future hedgehog safety tests in case they are more likely to come into physical contact with robotic lawn mowers.

### 4.3. Potential Biases in the Test Designs

When working with live animals, especially wildlife, it is often challenging to obtain a balanced experimental test design, as the distribution of, for example, the sex and age of the individuals being tested depend on factors such as the season, as well as chance. To prioritise animal welfare and minimise the stress caused to the hedgehogs included in the tests, by eliminating factors such as capture, transport, and handling by strangers, all of the tests took place at a single wildlife rehabilitation centre, where the hedgehogs had been in care and were considered ready for release back into the wild. We conducted the experiments on three occasions, limiting the availability and selection of individuals for the study, ultimately, resulting in a somewhat unbalanced composition of the 50 hedgehogs tested (15 adults and 35 independent juveniles; 30 females and 20 males). This should be taken into consideration when interpreting the results of our tests. 

As the novel object was covered in fabric and had permanent electronic parts distributed throughout, the plush toy was nonwashable. It was, therefore, decided that it could not be cleaned between the tests. This might have caused the odours of hedgehogs previously exposed to and exploring the novel object to accumulate, potentially rendering the novel object more stimulating for the last individuals tested, as a few cases of self-anointing were observed in the vicinity of the novel object [42]. The same applied to the cardstock paper covering the floor of the novel arena, as it remained the same throughout the tests. However, no hedgehogs defecated on the floor, and any nesting material or other foreign objects were removed in between the tests. As we decided to exclusively base our categorisation of shyness–boldness on the distribution of the individuals entering the arena or staying in the nest box for the duration of the 15 min test, we assumed that these factors (i.e., the novel object and the floor cover being more olfactory stimulating for the last individuals tested) likely did not influence the outcome of the tests. 

During the encounter tests with the robotic lawn mower, it was also not possible to clean the designated spot where each individual was placed, which potentially caused the area to become more olfactorily stimulating for the last individuals tested. This could, in principle, have influenced the behaviour of the hedgehogs tested. However, we did not notice any excessive sniffing activity from the hedgehogs directed at the ground where the individuals were placed. The hedgehogs, when deciding to expose their noses, away from the original position with their heads folded inwards, investigated their surroundings by smelling the air with the noses pointing upwards and not pointed in the direction of the ground. 

The wildlife rehabilitation centre and its surrounding garden, where the tests took place, was situated in a residential area near Aarhus in Denmark. During the tests we observed variability in the sensory stimuli coming from the surroundings. Some nights had heavy rainfall, whilst other nights had a range of different anthropogenic noise disturbances. These factors were beyond our control but, nevertheless, constituted a realistic scenario for hedgehogs inhabiting suburban residential areas. To the best of our abilities, we tried to adjust the timing of the tests to reduce the influence of these disturbances on our results.

It has been suggested that confidently determining personality in individuals requires repeated measures to determine whether these personality traits remain consistent during different test scenarios [66]. As we prioritised an experimental design to reduce the duration of the tests and handling of the hedgehogs to reduce stress [45] and allowing for the hedgehogs to be released into the wild as fast as possible, it was not possible to accommodate more repeated measures in our test design. It would be relevant to consider a method to increase the repeated measures of personality extending beyond the combined novel object and novel arena test and the encounter tests with a robotic lawn mower. 

Previous traumatic experiences may have influenced the reactions of the individual hedgehogs during the tests. However, the lack of knowledge about the life history of the hedgehogs prior to admission to the wildlife rehabilitation centre prevents any meaningful analysis of the influence of different traumas on the outcomes of the personality and encounter tests. 

### 4.4. Next Steps 

Knowing that certain models of robotic lawn mowers may cause injuries or even kill hedgehogs, understanding the extent of the problem is critical for hedgehog conservation and welfare. Therefore, we encourage the establishment of an open access international hedgehog database that can function as a daily record-keeping system for hedgehog carers, gathering and storing vital information on the hedgehogs coming into care. Additionally, collecting photographic evidence of hedgehogs injured or killed by electronic garden tools, such as trimmers and robotic lawn mowers, in order to quantify, document, and describe the types of damage caused by these machines is important (Berger et al. in prep.). 

On the basis of the present behavioural study, through the knowledge gained on how live hedgehogs position themselves when confronted by an approaching robotic lawn mower, it is now possible to prepare a realistic framework for a standardised safety test to measure the impacts of robotic lawn mowers on hedgehogs. Furthermore, complete standardisation requires the use of hedgehog crash test dummies, which can be 3D printed and applied by manufacturers of robotic lawn mowers in the process of designing and testing prototypes of new and more hedgehog-friendly machines. Ultimately, hedgehog crash test dummies should be used as proxies for dead hedgehogs in the standardised safety test and be designed to mimic reality and yield the same results compared to tests on dead hedgehogs.

The final step is to have the standardised hedgehog safety test implemented in the CENELEC protocol [41], testing and approving robotic lawn mowers for sale on the European market. This test would, furthermore, allow for a labelling system for hedgehog-safe robotic lawn mowers to be established, guiding the consumers to make the hedgehog-friendly choice when purchasing these tools. 

Work is currently well underway to achieve these described goals. But, for now, our advice remains to restrict the running of robotic lawn mowers to daylight hours and to check lawns for any wildlife species that may be vulnerable to an encounter with a robotic lawn mower, before turning on the machine. 

## 5. Conclusions

The robotic lawn mower market is growing rapidly, and, consequently, it is essential to help inform manufacturers on how to design more hedgehog-friendly machines in the future if we wish to eliminate this potentially negative influence on hedgehog survival in our backyards. This study sought to explore the personality and reactions of live hedgehogs when facing a disarmed, approaching robotic lawn mower to inform future standardised hedgehog safety tests allowing manufacturers to evaluate the performance of the new models being designed and developed at their facilities. 

In testing the reactions of hedgehogs towards an approaching (disarmed) robotic lawn mower, we conclude that that personality did not appear to affect the outcome; that adult and, thereby, more experienced hedgehogs tended to react in a more timid manner; and that the hedgehogs generally acted less boldly during their second encounter with the robotic lawn mower. 

The important insights gained from this study will be applied in the process of testing and refining the design of a hedgehog crash test dummy to be used in a future, standardised hedgehog safety test, which will be informed by the present results. This test will eventually serve to produce and approve hedgehog-friendly robotic lawn mowers and, ultimately, improve hedgehog conservation.

## Data Availability

All relevant data from this research have been made available in the publication.

## Appendix A

**Table A1:** Results from the encounter tests with a disarmed robotic lawn mower. “Personality” refers to the categories shy = 0 and bold = 1. Test number refers to the order of the two tests on each individual. Light describes whether the headlights of the robotic lawn mower were turned on or off during the specific test. Response denotes the behavioural response category of the individual, as defined in Section 2, “Materials and Methods”, with category 1 being the boldest and category 5 being the shyest.

Individual	Personality	Age	Sex	Weight (Gram)	Date	Time	Test Number	Lights	Response
Hedgehog 1	1	Adult	Female	1154	03.06.2022	23:17	Two	Off	3
Hedgehog 1	1	Adult	Female	1154	03.06.2022	23:15	One	On	4
Hedgehog 2	1	Adult	Female	1071	04.06.2022	00:00	One	Off	3
Hedgehog 2	1	Adult	Female	1071	04.06.2022	00:02	Two	On	3
Hedgehog 3	0	Adult	Male	774	04.06.2022	01:07	One	On	4
Hedgehog 3	0	Adult	Male	774	04.06.2022	01:10	Two	Off	4
Hedgehog 4	0	Juvenile	Female	497	04.06.2022	01:33	One	Off	3
Hedgehog 4	0	Juvenile	Female	497	04.06.2022	01:35	Two	On	3
Hedgehog 5	0	Adult	Female	996	04.06.2022	02:09	One	On	4
Hedgehog 5	0	Adult	Female	996	04.06.2022	02:13	Two	Off	4
Hedgehog 6	0	Adult	Female	924	04.06.2022	23:05	One	Off	4
Hedgehog 6	0	Adult	Female	924	04.06.2022	23:07	Two	On	4
Hedgehog 7	1	Adult	Female	1137	04.06.2022	23:40	One	On	3
Hedgehog 7	1	Adult	Female	1137	04.06.2022	23:42	Two	Off	4
Hedgehog 10	0	Adult	Female	772	05.06.2022	01:32	Two	On	1
Hedgehog 10	0	Adult	Female	772	05.06.2022	01:30	One	Off	3
Hedgehog 8	0	Adult	Female	645	05.06.2022	00:17	One	Off	3
Hedgehog 8	0	Adult	Female	645	05.06.2022	00:19	Two	On	3
Hedgehog 9	1	Adult	Female	699	05.06.2022	00:55	One	On	3
Hedgehog 9	1	Adult	Female	699	05.06.2022	00:56	Two	Off	3
Hedgehog 11	0	Adult	Male	1225	06.09.2022	21:03	Two	Off	3
Hedgehog 11	0	Adult	Male	1225	06.09.2022	21:01	One	On	4
Hedgehog 12	0	Adult	Male	1361	06.09.2022	21:37	One	Off	3
Hedgehog 12	0	Adult	Male	1361	06.09.2022	21:38	Two	On	3
Hedgehog 13	1	Juvenile	Female	361	06.09.2022	22:02	One	On	1
Hedgehog 13	1	Juvenile	Female	361	06.09.2022	22:06	Two	Off	3
Hedgehog 14	1	Juvenile	Male	381	06.09.2022	22:57	Two	On	2
Hedgehog 14	1	Juvenile	Male	381	06.09.2022	22:56	One	Off	3
Hedgehog 15	1	Juvenile	Male	328	06.09.2022	23:23	One	On	1
Hedgehog 15	1	Juvenile	Male	328	06.09.2022	23:27	Two	Off	2
Hedgehog 16	1	Juvenile	Female	361	06.09.2022	23:57	One	Off	1
Hedgehog 16	1	Juvenile	Female	361	06.09.2022	23:58	Two	On	1
Hedgehog 17	1	Juvenile	Female	328	07.09.2022	00:27	One	On	3
Hedgehog 17	1	Juvenile	Female	328	07.09.2022	00:30	Two	Off	3
Hedgehog 18	1	Juvenile	Male	216	07.09.2022	00:58	One	Off	3
Hedgehog 18	1	Juvenile	Male	216	07.09.2022	01:02	Two	On	3
Hedgehog 19	1	Juvenile	Female	321	07.09.2022	01:35	One	On	2
Hedgehog 19	1	Juvenile	Female	321	07.09.2022	01:35	Two	Off	2
Hedgehog 20	1	Juvenile	Female	264	07.09.2022	02:05	One	Off	1
Hedgehog 20	1	Juvenile	Female	264	07.09.2022	02:06	Two	On	4
Hedgehog 21	0	Juvenile	Female	318	07.09.2022	02:34	One	On	2
Hedgehog 21	0	Juvenile	Female	318	07.09.2022	02:36	Two	Off	3
Hedgehog 22	1	Juvenile	Female	343	07.09.2022	21:07	One	On	2
Hedgehog 22	1	Juvenile	Female	343	07.09.2022	21:10	Two	Off	2
Hedgehog 23	0	Juvenile	Male	383	07.09.2022	21:39	One	Off	1
Hedgehog 23	0	Juvenile	Male	383	07.09.2022	21:41	Two	On	3
Hedgehog 24	0	Juvenile	Male	398	07.09.2022	22:10	One	On	1
Hedgehog 24	0	Juvenile	Male	398	07.09.2022	22:11	Two	Off	1
Hedgehog 25	1	Juvenile	Female	308	07.09.2022	22:45	One	Off	1
Hedgehog 25	1	Juvenile	Female	308	07.09.2022	22:45	Two	On	4
Hedgehog 26	1	Juvenile	Male	387	07.09.2022	23:11	One	On	3
Hedgehog 26	1	Juvenile	Male	387	07.09.2022	23:13	Two	Off	3
Hedgehog 27	1	Juvenile	Female	383	07.09.2022	23:43	One	Off	1
Hedgehog 27	1	Juvenile	Female	383	07.09.2022	23:43	Two	On	4
Hedgehog 28	1	Juvenile	Female	231	08.09.2022	00:11	One	On	1
Hedgehog 28	1	Juvenile	Female	231	08.09.2022	00:13	Two	Off	2
Hedgehog 29	1	Juvenile	Male	221	08.09.2022	00:41	One	Off	1
Hedgehog 29	1	Juvenile	Male	221	08.09.2022	00:42	Two	On	1
Hedgehog 30	1	Juvenile	Male	196	08.09.2022	01:11	One	On	3
Hedgehog 30	1	Juvenile	Male	196	08.09.2022	01:12	Two	Off	3
Hedgehog 31	0	Adult	Male	1301	15.09.2022	20:34	One	On	2
Hedgehog 31	0	Adult	Male	1301	15.09.2022	20:35	Two	Off	4
Hedgehog 32	0	Juvenile	Female	230	15.09.2022	21:06	Two	On	1
Hedgehog 32	0	Juvenile	Female	230	15.09.2022	21:05	One	Off	2
Hedgehog 33	0	Adult	Female	718	15.09.2022	21:34	One	On	1
Hedgehog 33	0	Adult	Female	718	15.09.2022	21:36	Two	Off	2
Hedgehog 34	1	Juvenile	Female	322	15.09.2022	22:05	One	Off	2
Hedgehog 34	1	Juvenile	Female	322	15.09.2022	22:07	Two	On	2
Hedgehog 35	1	Juvenile	Male	300	15.09.2022	22:35	One	On	1
Hedgehog 35	1	Juvenile	Male	300	15.09.2022	22:36	Two	Off	2
Hedgehog 36	1	Juvenile	Male	316	15.09.2022	23:05	One	Off	1
Hedgehog 36	1	Juvenile	Male	316	15.09.2022	23:07	Two	On	2
Hedgehog 37	0	Juvenile	Female	298	15.09.2022	23:37	Two	Off	1
Hedgehog 37	0	Juvenile	Female	298	15.09.2022	23:35	One	On	2
Hedgehog 38	0	Adult	Female	668	16.09.2022	00:05	One	Off	3
Hedgehog 38	0	Adult	Female	668	16.09.2022	00:07	Two	On	3
Hedgehog 39	1	Adult	Female	1529	16.09.2022	00:35	One	On	4
Hedgehog 39	1	Adult	Female	1529	16.09.2022	00:36	Two	Off	4
Hedgehog 40	0	Juvenile	Female	318	16.09.2022	01:06	One	Off	2
Hedgehog 40	0	Juvenile	Female	318	16.09.2022	01:07	Two	On	3
Hedgehog 41	0	Juvenile	Male	236	16.09.2022	01:35	One	On	2
Hedgehog 41	0	Juvenile	Male	236	16.09.2022	01:36	Two	Off	2
Hedgehog 42	0	Juvenile	Male	213	16.09.2022	20:36	One	Off	3
Hedgehog 42	0	Juvenile	Male	213	16.09.2022	20:37	Two	On	3
Hedgehog 43	0	Juvenile	Male	478	16.09.2022	21:01	One	Off	1
Hedgehog 43	0	Juvenile	Male	478	16.09.2022	21:02	Two	On	2
Hedgehog 44	0	Juvenile	Male	226	16.09.2022	21:32	One	Off	3
Hedgehog 44	0	Juvenile	Male	226	16.09.2022	21:33	Two	On	5
Hedgehog 45	0	Juvenile	Female	326	16.09.2022	22:02	One	On	3
Hedgehog 45	0	Juvenile	Female	326	16.09.2022	22:04	Two	Off	3
Hedgehog 46	0	Juvenile	Male	413	16.09.2022	22:32	One	Off	3
Hedgehog 46	0	Juvenile	Male	413	16.09.2022	22:33	Two	On	3
Hedgehog 47	0	Juvenile	Female	302	16.09.2022	23:02	One	On	3
Hedgehog 47	0	Juvenile	Female	302	16.09.2022	23:03	Two	Off	3
Hedgehog 48	0	Juvenile	Female	295	16.09.2022	23:33	One	Off	3
Hedgehog 48	0	Juvenile	Female	295	16.09.2022	23:35	Two	On	3
Hedgehog 49	0	Juvenile	Female	300	17.09.2022	00:03	One	On	1
Hedgehog 49	0	Juvenile	Female	300	17.09.2022	00:04	Two	Off	3
Hedgehog 50	0	Juvenile	Male	168	17.09.2022	00:33	One	Off	3
Hedgehog 50	0	Juvenile	Male	168	17.09.2022	00:34	Two	On	3
